# Examining Cost Measurements in Production and Delivery of Three Case Studies Using E-Learning for Applied Health Sciences: Cross-Case Synthesis

**DOI:** 10.2196/13574

**Published:** 2019-06-04

**Authors:** Edward Meinert, Abrar Alturkistani, Kimberley A Foley, David Brindley, Josip Car

**Affiliations:** 1 Department of Primary Care and Public Health Digital Global Health Unit Imperial College London London United Kingdom; 2 Department of Paediatrics Healthcare Translation Research Group University of Oxford Oxford United Kingdom

**Keywords:** education, distance education, professional education, online education, online learning, costs and cost analysis, economics

## Abstract

**Background:**

The World Health Report (2006) by the World Health Organization conveys that a significant increase is needed in global health care resourcing to meet the current and future demand for health professionals. Electronic learning (e-Learning) presents a possible opportunity to change and optimize training by providing a scalable means for instruction, thus reducing the costs for training health professionals and providing patient education. Research literature often suggests that a benefit of e-Learning is its cost-effectiveness compared with face-to-face instruction, yet there is limited evidence with respect to the comparison of design and production costs with other forms of instruction or the establishment of standards pertaining to budgeting for these costs.

**Objective:**

To determine the potential cost favorability of e-Learning in contrast to other forms of learning, there must first be an understanding of the components and elements for building an e-Learning course. Without first taking this step, studies lack the essential financial accounting rigor for course planning and have an inconsistent basis for comparison. This study aimed to (1) establish standard ingredients for the cost of e-Learning course production and (2) determine the variance instructional design has on the production costs of e-Learning courses.

**Methods:**

This study made use of a cross-case method among 3 case studies using mixed methods, including horizontal budget variance calculation and qualitative interpretation of responses from course designers for budget variance using total quality management themes. The different implementation-specific aspects of these cases were used to establish common principles in the composition of budgets in the production and delivery of an applied health professional e-Learning course.

**Results:**

A total of 2 case studies reported significant negative budget variances caused by issues surrounding underreporting of personnel costs, inaccurate resource task estimation, lack of contingency planning, challenges in third-party resource management, and the need to update health-related materials that became outdated during course production. The third study reported a positive budget variance because of the cost efficiency derived from previous implementation, the strong working relationship of the course project team, and the use of iterative project management methods.

**Conclusions:**

This research suggests that the delivery costs of an e-Learning course could be underestimated or underreported and identifies factors that could be used to better control budgets. Through consistent management of factors affecting the cost of course production, further research could be undertaken using standard economic evaluation methods to evaluate the advantages of using e-Learning.

## Introduction

### Rationale

The World Health Report (2006) by the World Health Organization (WHO) [1] conveys that a significant increase is needed in global health care resourcing to meet the current and future demand for health professionals. Current challenges to health care resourcing include the increasing demand resulting from the aging population’s need for chronic disease management, in addition to the growing population placing an increased demand on primary care [2]. This increased demand on resources requires a scalable means to train resources; opportunities to optimize training through alternatives to face-to-face instruction present the possibility of increasing the pace and breadth of education to health care resourcing. A 2015 WHO systematic review of e-Learning for undergraduate health professional education concluded that “computer-based and Web-based e-Learning is no better and no worse than face-to-face learning with regards to knowledge and skill acquisition” [3]. e-Learning is defined as “an approach to teaching and learning, representing all or part of the educational model applied, that is based on the use of electronic media and devices as tools for improving access to training, communication, and interaction and that facilitates the adoption of new ways of understanding and developing learning” [4]. It presents a possible opportunity to change and optimize training in health professions (including clinical, allied, and applied health sciences, as well as patient education) by providing a scalable means for instruction, thus reducing the costs necessary in delivery and implementation. If we accept that pedagogically e-Learning can result in a positive educational effect used under optimal circumstances, which is still subject to ongoing investigation, there remains the possibility that deployment of e-Learning could affect the scale, cost, and reach of health professions education.

### Research Problem

One of the motivations for implementing e-Learning is the potential long-term efficiency gain in its delivery model [5,6]. A course delivered digitally versus the cost of a lecturer providing face-to-face instruction appears to have long-term cost favorability [7]. The literature often suggests that a benefit of Web-based learning is its cost-effectiveness compared with face-to-face instruction [8]; however, there is limited evidence validating comparison with other forms of instruction or standards for the budgeting of the costs in the production and execution of e-Learning courses. In the case of massive open online courses (MOOCs), there is limited evidence on the costs associated with their production [9]. In addition, the costs to develop an e-Learning course are significant when executed to a high standard. Although there are studies that capture data relating to factors associated with educational costs, measurement in these studies are collected inconsistently and include a wide variety of factors [3,10]. There is limited transparency in costing models because of sensitivity on where direct costs should be applied [11]. A systematic means is required to comprehensively record costs that can then subsequently enable testing of whether the e-Learning course has desirable economic properties and under what scenarios [12]. If proven so, this could assist in addressing the high cost of delivering health professions education. By contrast, should evidence point the other way, having discrete data points will allow those involved in online health education to identify ways to optimize costs in delivery. The primary issue here is identification of the direct and indirect costs in implementation, which then allows the execution of further economic evaluation.

### Aims and Objectives

This aim of this study was to establish an approach for identifying costs in the design, development, and deployment of applied health (defined as applied health subjects) sciences e-Learning courses and to subsequently propose a budgeting framework for the planning and management of e-Learning course implementations. The costs in this study include the direct and indirect costs from inception through course delivery. This approach will allow course designers and implementers to leverage knowledge gained from the study’s e-Learning case studies across different implementation contexts to better plan and manage future implementations, which will also create a reusable framework to apply cost planning. This work will demonstrate the effect in pre-implementation budget management against the proposed framework and should result in better course planning.

The study’s objectives are as follows:

- Establish an approach to capture standard components or ingredients for the cost of the production of an e-Learning course.
- Determine the effect that instructional design has on the production costs of e-Learning courses.

The study’s aims and objectives intend to address a gap in the research literature concerning implementation details on planning and executing e-Learning in health professions education [8]. In addition to limited cost-centered studies on e-Learning for health professions education, there are limited details on how course designers and producers are calculating the associated costs for production of these course types. Developing models will allow for the adoption of data sharing and course planning for improved management in execution of this course method and for further refinement and analysis. To explore this issue, this research examines the following 3 distinct e- Learning implementations as case studies.

#### Educating Administrative Staff to Engage With Young Patients

The course was created as a small private online course (SPOC) to prepare general practice administrative staff for issues in the management of adolescents. The course used case studies to provide training to help general practice staff feel confident in helping adolescents with a goal of improving the patient experience.

#### The Impact of Climate Change on Public Health

This course was created as an MOOC to educate citizens on the relationship between climate change and public health by using a multidisciplinary academic framework in data science to analyze, interpret, and present evidence. Core case studies focused on climate change and its health economic effect on local, regional, and national health systems.

#### Data Science in Health Care Using Real-World Evidence

This course was created as a blended MOOC to make learners aware of the effect data science can have on medicine and inspire the application of these methods across various undergraduate curriculum disciplines, the UK National Health Service commissioning support organizations, health care regulation organizations, and life sciences industries (ie, pharmaceuticals, biotechnology, and medical devices).The implementation of the blended MOOC was executed as a face-to-face course for learners; learners first took part in the MOOC and were then offered a residential course examining case studies. The target audience of the MOOC was allied health professionals or citizens looking to transition or enhance skills in data science in health care–related industries such as the pharmaceutical industry or biotech organizations. One of the key objectives of the course was to establish a global network of people to continue and advance the dialogue on data science in health care. Some of the course outcomes include the use and application of real-world evidence data collection and analysis techniques in health care settings.

## Methods

A mixed-methods case study design was selected to support a systematic means of observing the subject of investigation [13] and the ability to combine quantitative and qualitative approaches [14]. Mixed-methods research presents an opportunity to combine the strengths of quantitative and qualitative research to counteract the limitations inherent when each method is used in isolation [14]. In this study, for example, the limitations of quantitatively isolating cost differences in the 3 cases are strengthened by the repeatable and generalizable nature of the qualitative approach used to interpret results. Case studies were selected based on their relevance to the study inquiry and the ability to capture, record, and analyze data from each case. Each study was structured through a study protocol to govern the case execution.

### Case Study Overview

#### Case Overview

The objective of the case study is to inform the way future costs are budgeted in the development of e-Learning courses. The research forms part of a broader investigation into the costs associated with e-Learning course production; the main focus of each case was to collect primary evidence in the construction of these costs to allow for further research comparing results with other Web-based learning implementation types.

- Study question: how are the total costs for the production and delivery of an e-Learning course (dependent on type) calculated?
- Proposition: actual and budgeted costs will vary in the production or delivery of this course type.

Existing research literature indicates challenges in the capture of total costs for the production of Web-based learning despite standard methods for cost calculation [8]. The reason for this variance is likely because the skills required to create instructional learning design and to capture costs are different, and educators are not trained in cost accounting methods.

The analytical framework for this investigation is based on the cost analysis methods underpinning education economic evaluation developed by Levin [15], which extends the standard costing and variance calculation principles of activity-based costing [16-18]. The *ingredients method* [15] is used to capture total cost production against cost categories. It examines the core composition of costs in the delivery of an education intervention; this is an activity-based costing approach that seeks to understand the core components required for delivery. Defining core costs is critical to performing further economic evaluations, though it is important to note that the scope of this research is limited to cost identification and not further economic analysis (eg, cost-benefit analysis, cost-effectiveness analysis, cost-utility analysis, and cost-feasibility analysis).

Case study protocols (Multimedia Appendices 1,2, and 3,) were developed at study commencement to demonstrate the way costs would be captured and analyzed. These protocols, in addition to a protocol for qualitative and quantitative analysis of learning effect (which is outside the scope of the cost investigation) [19] were drafted, submitted, peer reviewed, and approved.

#### Data Collection Procedures

#### Evidence to Be Expected

To validate the costs reported in the actual budget (which was an actual cost report), at least 2 separate sources confirming the final reported amount were sought (eg, for a reported incurred cost for staff, timesheets were reviewed to match hours to costs, task completion, and assignment in a project plan). These data comparisons increased the likelihood that reported data were accurate.

#### Events to Be Observed

Although the course implementation was observed and additional studies completed investigating the education effect, the scope of this study was centered on the cost decision making, and the way production affected cost delivery. Therefore, the observation scope for this study focused on reported costs and the way these correlated data to time actuals.

#### Documentation to Be Reviewed

The project budget, actual costs, and timesheets were reviewed for this study. Although there will be a review of the completed course and observation of the way the course uptake is completed, the latter shall be excluded from this study. A traceability log was maintained in Microsoft Excel linking the research questions to data sources and the study findings.

#### Protocol Questions

Study question: how are the total costs for the production and delivery of an e-Learning (type dependent on implementation type) course calculated?

- The costs will be measured and ingredients captured and analyzed to understand the factors affecting course production.
- Data will be collected to support the cost analysis categories.
- The corresponding evidence will be used to summarize ways that cost capture practices could be improved.

### Study Framework

#### Plan

Each case study followed a 6-stage process in the investigation (Table 1) [13]. The research question centered on identifying the total costs of production and delivery in these e-Learning implementations, and the effect of factors on variance from anticipated budgets. It was selected because evidence from the literature suggests inconsistency in the determination of costs for the delivery of Web-based courses [20]. This is significant because the lack of consistent cost capture mechanisms for Web-based learning compromises any further evaluation. Despite available methods to avoid this outcome, the literature presents research with claims that Web-based learning is more *cost effective* than face-to-face learning. This research provides a structured means to generate evidence to subsequently evaluate such claims by collecting baseline data on course production for further evaluation.

#### Design

The research design (Table 2) was structured on 4 components (proposition, the case definition, logic linking data to the proposition, and criteria for interpreting findings) to explore the following research question: how are the total costs for the production and delivery of e-Learning calculated (with the e-Learning implementation type variant depending on the case study)? Given the inconsistency in the presentation of costs in the literature and recognizing that using budgets to determine the cost of educational delivery is insufficient [21], the governing proposition of the investigation was that there would be variance between the budgeted costs and the actual costs to produce the course. This was explored through cases that would examine the cost and the measurement of costs and place value on ingredients. Levin developed this *ingredients method* to capture and analyze the costs in the delivery of an educational program. To link the case to the proposition, the cost calculation was completed and then interpreted via a variance calculation of actual to budgeted costs, and rationales were developed to justify variations.

Examination of these cases provides data to analyze the relationship between course production and budgeting in the delivery of e-Learning and provides evidence for constructing accurate budget models.

Each case was tested for construct validity (testing that data sources come from multiple sources), external validity (testing that demonstrates how principal findings could be extensible) and reliability (testing that shows how the activities of the study can be replicated) to ensure data triangulation, the ability for study replication, and standardization for project data collection [13]. Ethical approval for each study was obtained through the Imperial College Education Ethics Research Committee (case 1: EERP1516-005; case 2 and 3: EERP1617-030).

#### Prepare

The investigation was focused on cost measurement and analysis, structured by 3 cost categories, and further subdivided using a 7-step process (illustrated in Table 3 below) to analyze the pre- and postproduction budget [21]. Levin’s model uses an activity-based standard-costing accountancy approach, which assigns costs as they are consumed per implementation area [25,26].

**Table 1:** Case study framework*.*

Stage	Outcome
Plan	Case description and linking of case approach to investigation outcomes.
Design	Construction of research design and linkage of research questions, data, and criteria for evaluation and synthesis.
Prepare	Draft, execution, and approval of study protocols.
Collect	Data collection strategy executed from a *realist* perspective to capture the decision making of the course designers centered on cost attributes.
Analyze	Data extracted into categories for review and analyzed for variance calculation. Data analysis centers on 3 cost categories in the design of the preproduction budget submitted to the funder for each case. Category A: concept and measurement of costs: The preproduction budget was analyzed for the following ingredient categories: (1) personnel, (2) estate charges, (3) equipment and materials, (4) indirect costs, and (5) stakeholder costs; Category B: placing values on ingredients: With the full cost of production defined, values were associated with each ingredient subcategory to reflect the chargeable cost; Category C: calculating costs: To record a variance calculation, a comparison of the budget with the incurred costs was reviewed on a quarterly basis. Variance=Actual spending–Budgeted spending.
Share	The findings of the variance calculation and synthesis of analysis of reasons leading to variation were presented in a report for publication in a peer-reviewed journal. (This study).

**Table 2:** Case study research design*.*

Case (year)	Study question	Proposition	The case (definition)	Logic linking data to the proposition	Criteria for interpreting findings
Case 1: Educating administrative staff to engage with young patients (2016) [22]	How are the total costs for the production and delivery of this e-Learning course calculated?	Actual and budgeted costs will vary in the production/delivery of this course type	Determination and measurement of costs	Cost analysis of project, actual, and underreported costs	Variance calculation from the project budget
Case 2: The impact of climate change on public health (2017) [23]	How are the total costs for the production and delivery of this e-Learning course calculated?	Actual and budgeted costs will vary in the production/delivery of this course type	Determination and measurement of costs	Cost analysis of project, actual, and underreported costs	Variance calculation from the project budget
Case 3: Data science in healthcare using real world evidence (2018) [24]	How are the total costs for the production and delivery of this e-Learning course calculated?	Actual and budgeted costs will vary in the production/delivery of this course type	Determination and measurement of costs	Cost analysis of project, actual, and underreported costs	Variance calculation from the project budget

**Table 3:** Course production ingredients cost analysis.

Cost categories	Objectives—adapted from Levin (2001, 2018) [27,21]
Category A: concept and measurement of costs	Steps 1 to 5: Describe the concept of costs; show the inadequacy of budgets for cost analysis; present a methodology for measuring costs; identify categories of cost ingredients; describe sources of cost information
Category B: placing values on ingredients	Steps 6 and 7: Describe the purpose and principles for determining the values of ingredients; present methods for placing values on specific types of ingredients

#### Collect

Evidence from the course was retrieved from project documents and records of finance activity. The data collection strategy was executed from a realist perspective to capture the decisions made by the course designers; however, it did not incorporate a relativist perspective with regard to stakeholders, through further qualitative investigation. This decision was made to avoid interference in course delivery. To control biased selectivity and reporting bias, the data were sourced through multiple sources, including finance logs (and notes), data submitted to the employer, the funder, and timesheets. A traceability log was maintained linking the study questions to the relevant data sources and the study findings.

#### Analyze

Data analysis centered on the 3 cost categories and followed the 7-step process for cost definition.

##### Category A: Concept and Measurement of Costs

The preproduction budget was analyzed for the following ingredient categories: (1) personnel, (2) estate charges, (3) equipment and materials, (4) indirect costs and (5) stakeholder costs. The initial budgets did not reflect time for stakeholder costs (effort from third-party lecturers); therefore, this was captured as the additional time that was monitored in the study (and added for budget variance calculation), as there was no value for this in the data submitted to the funder.

##### Category B: Placing Values on Ingredients

With the full cost of production defined, values were associated with each ingredient subcategory to reflect the chargeable cost (including direct and indirect costs).

##### Category C: Calculating Costs

As each course was implemented in 1 year, and the courses were Web-based, there were no multiyear costs to calculate; the one-time cost of the project and the variance of the projected budget to the actual budget were the only variables under consideration. To accomplish this, the variance calculation of the budget to the incurred costs was undertaken at the completion of the project. The variance calculation compares actual costs to adjusted standard conditions based on occurrence [28].

The variance calculation formula is as follows: Variance = Actual spending − Budgeted spending.

##### Analyzing Costs of Observed Budget Variance Calculations

**Figure 1:**
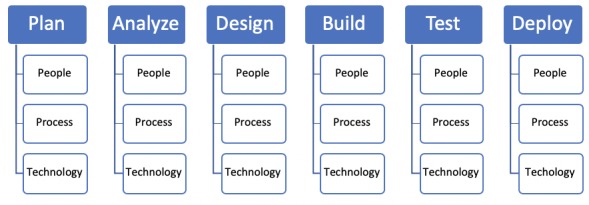
Isolating variance during project stage to total quality management criteria.

To determine the reasons for favorable or negative budget variance, the course designers were interviewed to determine the factors contributing to budget variance. This qualitative work was planned via the consolidated criteria for reporting qualitative research [29] to ensure that the appropriate trained staff conducted interviews, study design included the purposeful sampling of the course designers, sessions could be validated in the interviews, and the resultant analysis and findings would be repeatable [29]. The sessions were conducted as semistructured interviews transcribed and coded using thematic analysis [30] using total quality management (TQM) as coding criteria. TQM [31] is a quality appraisal method used to analyze factors affecting operational efficiency [32]. TQM provides a means to categorize issues relating to people, process, or technology through applying a systems approach to management (see Figure 1). For each area of cost variance, the course designers were asked to review budget reports to identify stages in the project lifecycle for variances in forecast and to describe the contributing factors. After the interview, these were coded independently by 2 researchers to create a novel means of interpreting the cost calculation variance. For example, if a cost variance was attributed to stakeholder costs, the researchers would examine reported quarterly budgets (or at the project time interval) and determine where the variance began occurring. If the variance commenced during the build stage of the project, the project plan was analyzed, and questions surrounding the activities of the project were asked of the course designers to determine the root cause.

The key themes for the TQM analysis are presented in each case indicating the summary perspective of areas for improvement or efficiency in e-Learning budget creation.

#### Share

The findings of the variance calculation and the deductive-inductive interpretation of reasons leading to variation were presented in a case report to the course design and production team. Feedback was gathered on analysis and results; the key findings for each report were prepared for publication for a peer-review journal.

### Cross-Case Synthesis

**Figure 2:**
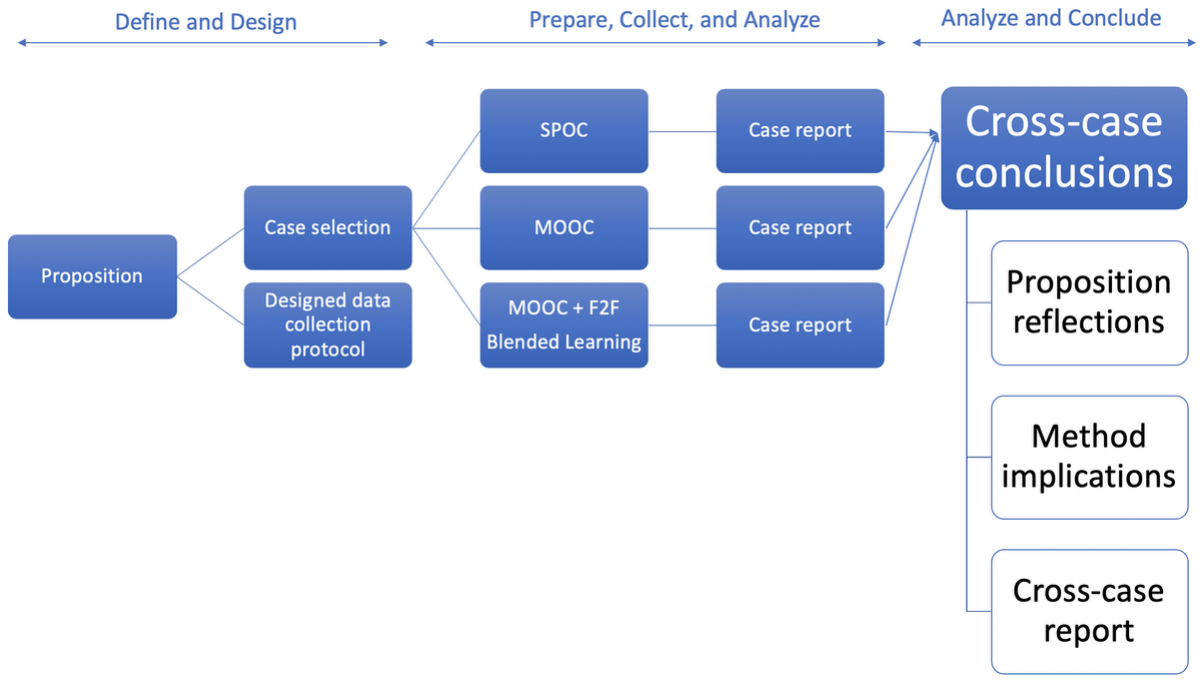
Cross-case synthesis. MOOC: massive open online course; SPOC: small private online course.

To derive results from the composite analysis of the cases, this study makes use of cross-case study synthesis [13] as illustrated in Figure 2. The standard variables in the cases are centered on ingredients and their incurred cost variance from budget.

## Results

### Course Production Costs

#### Category A: Concept and Measurement of Costs

Costs for each case were summarized into components and separated into ingredient cost categories (Table 4).

#### Category B: Placing Values on Ingredients

Upon completion of the analysis of the ingredients of the course production, initial budgets were created and submitted to the funder.

#### Category C: Analyzing costs

##### Budget Variance Calculation

###### Case 1

The project implementation costs, in this case, had a negative variance of 41% (Multimedia Appendix 4). The most significant negative variance (135%; Multimedia Appendix 4) was in equipment and materials, primarily from the costs of app development in the creation of a Web-based course. As the production team had not created a Web-based course before, there was a significant underestimation of the amount of time required to build and configure the system (which was developed using the Open edX learning management system platform) and complete course editing. In addition, specialist recording equipment had to be procured that was not understood at the time of budget completion. The next most substantial negative variance (76%; Multimedia Appendix 4) was the amount of time required from third-party stakeholders in the production of learning materials. The amount of time allocated for recording the lecturers was underestimated; there had to be several re-runs of the recordings to address content changes. The lowest negative cost variance (31%; Multimedia Appendix 4) was in the personnel costs to deliver the course. Although the variance was the smallest of the 3 categories, it was significant because the course production team did not receive any additional compensation for their additional work; this extra work was captured in the project timesheets but not submitted to the funder for reimbursement.

**Table 4:** Ingredient categories*.*

Ingredient categories	Cost components
Personnel	University staff
Estate charges	Information technology services charges
Equipment and materials	Course production equipment and application development costs for the creation of software to support the massive open online course
Indirect costs	University overheads
Stakeholder costs	Staff for third-party subject matter consultancy

###### Case 2

The actual costs varied from the budgeted cost in personnel, equipment and materials, and stakeholder costs, and the total cost of production has a negative variance of 113% (Multimedia Appendix 4) from the budgeted amount. The most significant variance was in stakeholder costs, where the total time for external lecturers and subject matter experts to deliver work was significantly underbudgeted, with a negative variance of 190% (Multimedia Appendix 4). The reason for this underestimate was that videos had to be reshot twice and the amount of time allocated to retrieve stakeholders and complete associated course updates dramatically affected the budget. The second largest variance was in personnel; the cost variance was directly related to the additional production time required for the video reshoots, in addition to the iteration of the development of the platform. The course implementation online learning provider also switched from edX to FutureLearn learning management system during the project, requiring rework of previously completed tasks. As the team was not experienced on the FutureLearn platform, this further accounted for additional effort and the unfavorable budget variance; a team with experience and training on design for the course material would most likely have attained different results. Finally, equipment and materials were also underestimated with a negative variance of 133% (Multimedia Appendix 4), having to do with additional software required for video editing and additional workstations gathered to deal with additional editing required in the course development.

###### Case 3

In contrast to the previous case studies, this case demonstrated a positive variance of 16% (Multimedia Appendix 4) from the initial budget. Stakeholder costs for subject matter expert lecturers were slightly overestimated but close to budget. It is important to note that the third-party stakeholder team had significant previous experience working together for producing related coursework, and this could have led to the precision in effort estimation. Equipment and materials had a significant positive variance of 37% (Multimedia Appendix 4); the reason for this is that not all the equipment planned for the course development was necessary because there was efficiency derived in the course production and streamlining of data science modules that were thought to have required custom app development. Personnel had a negative variance of 13%; this was related to additional effort required in video editing. In addition, the course was completed ahead of schedule and in less time than was anticipated.

The construction of the cost ingredients and subsequent cost analysis underwent 3 validation tests (Table 5).

Issues affecting budget variance were classified using TQM to categorize factors influencing the budget (Table 6). Although each course was implemented with a varying form of e-Learning, the issues affecting each case were similar and cross-applicable. The critical consideration in budgeting is less an aspect of the type of e-Learning, but more the planning associated with the project management of the creation of the course.

**Table 5:** Cross-case results validation tests*.*

Case	Construct validity	External validity	Reliability
1	To achieve data triangulation, the case study had multiple sources of cost data. (1) The project budget that was submitted to the project funder, (2) the actual costs submitted to the funder at the completion of the project, and (3) the timesheet log of hours captured by the course implementers. The final case report was reviewed, and feedback gathered from the course designers (BS, MT); any inconsistencies or inaccuracies were corrected.	By using Levin’s ingredients method for cost identification, the case followed an established costing procedure that is used as the basis for analytic frameworks for economic evaluation in education. This process based on a common analytic framework allows for the generalization of the study findings to similar use cases.	A study protocol was created at the commencement of the case; the protocol details the structure of the study and details how data were collected to ensure the reliability of the results.
2	Multiple sources of cost data and reporting data were used to validate that data sources were an accurate record of what occurred. (1) The project budget created at the project commencement, (2) the actual cost report submitted at the completion of the project, (3) the timesheet log of hours captured by each team resource, (4) a third-party work-log for course production and monitor of billable hours recorded charged to the program, (5) external audit reports on the course construction, and (6) review of notes from monthly reviews of budget spend. The final case report was reviewed, and feedback gathered from the course designers (BS, MT); feedback was provided and reviewed by the research team to ensure implementation accuracy.	The repetition of a model used in prior research [22], application of Levin’s ingredients method for education intervention analysis, and use of standard costing and variance calculation activity-based costing methods demonstrated a common analytic framework that is transportable to other studies.	To achieve this test, a study protocol was used and formed the governing basis for the study.
3	The data sources for each ingredient category were sourced from (1) the initial project budget, (2) reported submitted costs, (3) a time log of hours worked, and (4) a third-party work-log of the activities of subcontracted courses. The final case report was reviewed to ensure accuracy.	The same process that was used in the 2 previous cases was replicated [24], and application of Levin’s ingredients method for education intervention analysis demonstrated a common analytic framework transportable to other electronic learning studies.	A minor variation of the previous study protocols executed was used and stored as the governance framework for the study.

**Table 6:** Total quality management category of issues affecting budget adherence to the model.

Cases	Issue	People	Process	Technology
Case 1	The inadequacy of project budgets at the commencement of Web-based learning for new teams	—^a^	X^b^	X
	Underreporting of personnel costs	X	X	—
Case 2	Resource task estimation and management	—	X	—
	Contingency planning	—	X	—
	Third-party resource management	X	X	—
	Need for an update of course materials	—	X	X
Case 3	Cost efficiencies in the delivery of a course piloted in previous years	—	X	—
	Experience and relationship of the course learning team	X	—	—
	Agile project management methods and iterative budget management	—	X	—

^a^Not applicable.

^b^Applicable.

### Project Management

Each case implemented project management methods for the organization of crucial deliverables and tasks in their design and integrated learning design methodology in different ways. Case 1 employed project-related task-centered actions constructed to match each learning outcome. Case 2 integrated the analysis, design, development, implementation, and evaluation (ADDIE) model, and course planning was structured along each of these design stages, whereas case 3 implemented an agile project management model (with iterations) while using the ADDIE model in course construction.

### Participant Information

#### Case 1

A total of 124 learners enrolled in the SPOC from September 2016 to December 2016 (Table 7). Of these, 84% completed the course and received a postcourse certificate. The course uptake and completion, however, did not influence the production costs postcourse implementation as the course was designed as a self-managed SPOC not requiring further administration after deployment.

**Table 7:** Electronic learning implementation participation summary*.*

Case (year)	Learners, n	Completion, %
1: Educating administrative staff to engage with young patients (2016)	124	84
2: The impact of climate change on public health (2017) [19]	968	17
3: Data science in health care using real world evidence (2018)	5036	12

#### Case 2

A total of 968 learners participated in the MOOC from November 2017 to December 2017 (Table 7). Of these, 17% completed the course. The course completion ratio was in line with completion rates for MOOCs [33], where although there is a high uptake of initial learners, completion of course activity ranges from 8% to 20%.

#### Case 3

A total of 5036 learners participated in the MOOC from September 2018 to December 2018 (Table 7). Of these, 12% completed the course. The course completion ratio was also in line with completion rates for MOOCs [33]. A blended residential course was held in November 2018, with the participation of 14 learners (these learners were inclusive in the MOOC set). In this residential course, the participants completed the MOOC as prelearning and then undertook case studies, putting course learning into practice.

## Discussion

### Principal Findings

This study aimed to establish an approach for identifying the costs in the design, development, and deployment of applied health professions e-Learning courses. The standard components for the construction of an e-Learning course were determined by the methods used in this study, which combined existing approaches for cost budgeting with qualitative methods for the interpretation of results. Although Levin’s ingredients method provides a mechanism for categorizing costs design and implementation costs for budgeting, TQM provides a qualitative framework to examine the effect of the design and production decisions on the budget. The key issues affecting the ability of the budget to deliver in line with expectations at the close of the project were related to process issues. Familiarization with technology was also a key issue in cases 1 and 2, where familiarity with production methods and learning technology had an effect on anticipated effort.

The key recommendations made from examination of these cases center on 3 areas of process-related enhancement, 1 having to do with project management and the remaining 2 having to do with budget management, both related to the course production and instructional design:

#### Project Management: Linkage of Instructional Design Method to Stages in the Project Lifecycle With Time Tracking

Project management enables the planning and prioritizing of activities; management of risk, issues, and actions; and ensuring quality. In these observed cases, the use of robust project management methods and the development of iterative methods to validate learning materials tended to create favorable results. In addition, linking an instructional design approach to project stages and tracking tasks by time to each component creates awareness and links the associated financial effect of delivery to course building.

#### Budget Planning: Use of Confidence Factors in Budget Time Estimating

A vital issue in all cases was overestimating the amount of effort required to build tasks. To better manage time tracking, we have suggested tracking task by time linked to learning design, but as an additional measure, building confidence factors into budgets allows a degree of error and contingency when building initial budgets. A confidence factor is a percentage of variance added to an initial cost forecast that can be added as a contingency; applying confidence factors based on requirements, the familiarity of approach, and other factors can lead to higher estimation precision.

#### Budget Planning: Modeling Budget Forecasting on Similar Implementations

Case 3 was the most successful in delivery because the course team had worked together delivering similar content, was able to gain efficiency in having preexisting relationships, and had an evidence base to build their cost models from. When planning e-Learning implementations, the starting point should similarly be previous projects or using data from the literature on factors influencing costs, so budgets are not determined from scratch. Part of the observed budget variance issues in cases 1 and 2 had to do with estimates for costs not built on prior evidence; this can be controlled by using an experience-driven starting point.

### Strengths and Limitations

This study analyzes 3 distinct cases of e-Learning covering 6128 applied health learners in 3 years and provided a comprehensive summary of the issues affecting the production and development of a course. This information could be useful for course designers in the planning of their e-Learning implementations and for drawing on lessons learned to plan budgets that ensure projects meet their objectives.

We noted 4 limitations with this study. Case study research can only provide a snapshot of activities as observed in each case, and there is a possibility that these cases may have limited applicability to other contexts. This has been mitigated using construct validity, external validity, and reliability tests in each case, but it is important to note that case study research has an inherent limitation in the observation of events under consideration due to the design; experimental methods deliver more rigorous results to test results. In addition, the selection of the case studies was opportunistic, as they were e-Learning projects accessible within the first author’s research unit. The second limitation is that further qualitative investigation of attitudes, views, and perceptions of stakeholders was not undertaken. This would have added an additional dataset to analyze factors affecting budgeting, meaning that the researchers drew conclusions from data that may have been viewed differently with further direct inquiry from stakeholders. It is important to note however, that stakeholders did review final case reports for accuracy and consistency with events. The third limitation is that the study did not undertake critical examination of the decisions made by the course designers in authoring tools, license costs, expertise, and other factors affecting the direct costs; examination of these costs including triangulation among the 3 sources would lead to further evidence affecting results. Finally, the study made use of a mixed-methods approach to analyze horizontal budget analysis but did not undertake an analysis for offsetting or magnifying variances, return on investment, forecasting, sensitivity analysis, or other financial planning and analysis methods. An economic study focused on outcomes and cost could provide further data that would potentially influence implementation considerations.

### Further Research

The outputs of this study, in addition to the process of execution and reflection on both strengths and limitations, suggest 3 possible areas for future research:

#### Standards for Costing Economic Evaluations of e-Learning Implementations

Limited economic evaluations are conducted on e-Learning, most likely because educators focus on content delivery and educational effect rather than creating cost evidence. This study has created an extension of existing costing methods and demonstrated how it can be applied to e-Learning, allowing future researchers to reuse this approach to create consistent costing data, which could be subsequently benchmarked. With a growing evidence base of e-Learning cost data, this could also promote further research into various forms of economic evaluation, to create possible business cases for future investment in e-Learning, should value be demonstrated.

#### Integration of Project Management, Instructional Design Methods, and Costing

This study observed benefits in the combination of project management methods and instructional design methods; further research investigating ways of adopting existing instructional design methods with project management methodologies and linking these methods with cost management approaches could help address the high investment cost required in e-Learning.

#### Cost and Value Perceptions of Students and Educators

Using improved cost data from the approaches in this research, further research could attempt to identify perceptions of cost and value by comparing the perspectives of students and educators.

### Conclusions

e-Learning research consistently refers to the promise and opportunity of its cost-effectiveness in contrast to face-to-face instruction; however, the underlying data supporting the costs necessary for their delivery are not well understood [8]. To implement further economic evaluation to understand proprieties demonstrating the value of e-Learning in contrast to other learning types, it is first necessary to develop a standard means of calculating costs in the delivery of these types of projects. Through consistent management of factors affecting costs in course production, further research could be undertaken using standard economic evaluation methods to evaluate the advantages of using e-Learning. This study enables an understanding of the issues affecting cost planning for the design, development, and deployment of e-Learning courses and also provides recommendations on controlling cost variance within e-Learning projects. This study contributes a systematic approach to costing in e-Learning that course designers and researchers could use to design and calculate costs in the production and deployment.

## Appendix

Multimedia Appendix 1 Case study protocol – educating administrative staff to engage with young patients.

Multimedia Appendix 2 Case study protocol – the impact of climate change on public health.

Multimedia Appendix 3 Case study protocol – data science in healthcare using real world evidence.

Multimedia Appendix 4 Ingredient costs variance calculation.

## Abbreviations

ADDIE: analysis, design, development, implementation, and evaluation
MOOC: massive open online course
SPOC: small private online course
TQM: total quality management
WHO: World Health Organization
