# Supplementary material for: Examining Cost Measurements in Production and Delivery of Three Case Studies Using E-Learning for Applied Health Sciences: Cross-Case Synthesis
Source: J Med Internet Res. 2019 Jun 4;21(6):e13574. doi: 10.2196/13574 (PMC6746105; doi:10.2196/13574)
Supplement: Multimedia Appendix 3 [file jmir_v21i6e13574_app3.docx]

# Multimedia Appendix 3: Case study protocol – Data Science in Healthcare using Real World Evidence

Structure adopted from Yin [13]

This study protocol is a modification of protocols previously developed for analysis for use in eLearning implementation [24].

**Section A. Overview of the Case Study**

1. Mission and goals reflecting the interest of the case study’s sponsor (if any) and audience

- The objective of the case study is to inform the way future costs would be budgeted in the development of online learning. The research forms part of a broader investigation into the costs associated with the production of online learning; the main focus of this report was to collect primary evidence in the construction of these costs to allow for further research comparing results with other online learning implementation types.

1. Case study questions and propositions

- Study question: How are the total costs for the production and delivery of a massive online course calculated? ( See Table C below)
- Proposition: Actual costs and budgeted costs will vary in the production/delivery of this course type.

The state of the literature indicates challenges in the capture of total costs for the production of online learning, despite standard methods for cost calculation [8]. The reason for this variance is likely because the skills required to create robust instructional learning design and to capture costs are different, and educators are not trained in cost accounting methods.

1. A theoretical framework for the case study essential readings

- The analytical framework for this investigation is based on cost analysis methods underpinning education economic evaluation developed by Levin [15], which extends standard costing and variance analysis principles of activity-based costing [16–18]. Defining core costs is critical to performing further economic evaluations, though it is important to note that the scope of this research is limited to cost identification and not further economic analysis (e.g. cost-benefit analysis, cost-effectiveness analysis, cost-utility analysis, cost-feasibility analysis)

Table C Cost Categories and their Objectives

| Cost Categories | Objectives |
| --- | --- |
| 1. Concept and measurement of costs | 1. Describe the concept of costs 2. Show the inadequacy of budgets for cost analysis 3. Present a methodology for measuring costs 4. Identify categories of cost ingredients 5. Describe sources of cost information |
| 1. Placing values on ingredients | 1. Describe the purpose and principles for determining the values of ingredients 2. Present methods for placing values on specific types of ingredients |
| 1. Analyzing costs | 1. Summarize the application of cost methodology with the use of a cost worksheet 2. Show how to analyze the distribution of cost burdens among different stakeholders 3. Address cost estimation for multiyear projects 4. Illustrate the estimation of costs under uncertainty 5. Present different ways of using costs for decisions |

1. Role of protocol in guiding the case study research

- The protocol was developed at study commencement to demonstrate the way costs would be captured and analyzed in the study. This protocol, in addition to a protocol for qualitative and quantitative analysis of learning effect [19], were drafted and submitted to peer review by the Imperial College Education Ethics Committee. The role of this protocol is to memorialize the intended methods, submit them to peer review to validate the research design, and serve as the framework for the investigation. Any deviations are to be documented and submitted for review.

**Section B. Data Collection Procedures**

1. Key stakeholders
   1. Research team: responsible for collecting field work
      1. EM – Lead researcher
      2. PS – Co-investigator
      3. TS – Co-investigator
   2. Course team: team observed in the case study
      1. KF – Research associate
      2. AA – Research assistant
      3. HC – Subject Matter Expert
      4. YE – Subject Matter Expert
      5. MT – Business analyst
2. Data collection plan (covers the type of evidence to be expected, including the roles of people to be interviewed, the events to be observed, and any documentation to be reviewed in the field)
   1. Evidence to be expected

Costs incurred in the production of the online course. This will be calculated using three different data sources to provide triangulation of results

- 1. Events to be observed

While the course implementation will be observed and additional studies completed investigating the education effect, the scope of this study is centered on the cost decision making, and the way production affected cost delivery. Therefore, the observation scope for this study will be focused on reported costs and the way these correlate data to time actuals.

- 1. Documentation to be reviewed

The project budget, actual costs, and timesheets will be reviewed for this study. While there will be a review of the completed course and observation of the way the course uptake is completed, the latter shall be excluded from this study. A traceability log will be maintained in excel linking the research questions to data sources and the study findings.

1. Expected preparation before fieldwork (identified specific information to be reviewed and issues to be covered before fieldwork)
   - - 1. Confirmation of the initial budget from the funder
       2. Confirmation of the appointment of the course delivery team
       3. Ethical approval

**Section C. Protocol questions**

1. Study question: How are the total costs for the production and delivery of a small private online course calculated? (Table C above)
   1. The costs shall be measured and ingredients captured and analyzed to understand the factors affecting course production
   2. Data shall be collected to support the cost analysis categories
   3. The corresponding evidence will be used to summarize ways cost capture practices could be improved

**Section D. Tentative Outline for the Case Study Report**

1. The audience for the report and stylistic preferences for communicating with the audience(s)
   1. The case report will be presented as a manuscript for publication in a peer-reviewed journal. The audience will be an academic audience with the intent to inform future practice for the development of online learning
2. Case Report Format
   1. The case report will be structured as a standard research report, covering an Introduction/rationale, Methods, Results, Discussion, and Key findings against a research question.
