# Supplementary material for: Examining Cost Measurements in Production and Delivery of Three Case Studies Using E-Learning for Applied Health Sciences: Cross-Case Synthesis
Source: J Med Internet Res. 2019 Jun 4;21(6):e13574. doi: 10.2196/13574 (PMC6746105; doi:10.2196/13574)
Supplement: Multimedia Appendix 4 [file jmir_v21i6e13574_app4.docx]

Multimedia Appendix 4: Ingredient costs variance calculation*.*

|  | Case 1 | | | | Case 2 | | | | Case 3 | | | |
| --- | --- | --- | --- | --- | --- | --- | --- | --- | --- | --- | --- | --- |
|  | Budget | Actual | Variance | Var  % | Budget | Actual | Variance | Var  % | Budget | Actual | Variance | Var % |
|  |  |  |  |  |  |  |  |  |  |  |  |  |
| Personnel | £71,119 | £93,455 | £22,336 | 31% | £43,646 | £88,456 | £44,810 | 103% | € 102,040.75 | € 115,432.00 | € 13,391 | 13% |
| Estate charges | £8,949 | £8,949 | £0 | 0% | £2,345 | £2,345 | £0 | 0% | € 12,625.00 | € 12,625.00 | € 0 | 0% |
| Equipment and materials | £16,773 | £39,455 | £22,682 | 135% | £3,255 | £7,599 | £4,344 | 133% | € 244,517.19 | € 153,432.00 | (€ 91,085) | -37% |
| Indirect costs | £22,717 | £22,717 | £0 | 0% | £11,725 | £11,725 | £0 | 0% | € 88,317.06 | € 88,317.00 | (€ 0) | 0% |
| Stakeholder costs | £9,823 | £17,333 | £7,510 | 76% | £25,999 | £75,332 | £49,333 | 190% | € 50,000.00 | € 48,342.00 | (€ 1,658) | -3% |
| *Total* | £129,382 | £181,910 | £52,527 | 41% | £86,970 | £185,457 | £98,487 | 113% | € 497,500.00 | € 418,148.00 | (€ 79,352) | -16% |
